# Integrated radiopathomics nomogram for predicting angiogenic microvascular patterns in NSCLC: a dual-center validation study

**DOI:** 10.1080/07853890.2026.2654291

**Published:** 2026-04-17

**Authors:** Ronghua Wang, Lin Wang, Xinzheng Wang, Shuai Quan, Linning E., Jiangfeng Du

**Affiliations:** ^a^ Department of Radiology, Shanxi Bethune Hospital, Shanxi Academy of Medical Sciences, Third Hospital of Shanxi Medical University, Tongji Shanxi Hospital, Taiyuan, China; ^b^ Department of Pathology, Shanxi Bethune Hospital, Shanxi Academy of Medical Sciences, Third Hospital of Shanxi Medical University, Tongji Shanxi Hospital, Taiyuan, China; ^c^ Department of Radiology, The People’s Hospital of Longhua, Shenzhen, China; ^d^ GE HealthCare, Shanghai, China; ^e^ Department of Radiology, Shanxi Provincial People’s Hospital, Fifth Hospital of Shanxi Medical University, Taiyuan, China; ^f^ Department of Medical Imaging, Shanxi Key Laboratory of Intelligent Imaging, First Hospital of Shanxi Medical University, Taiyuan, China

**Keywords:** Angiogenesis, computed tomography, non-small cell lung cancer, pathomics, radiomics

## Abstract

**Background:**

To develop and validate an integrated radiopathomics nomogram combining multiphase CT images, H&E-stained slides, and clinicopathological variables for predicting microvascular patterns (MVPs) in non-small cell lung cancer (NSCLC).

**Methods:**

We retrospectively included consecutive surgically resected NSCLC patients from two centers (*n* = 258). Patients from center 1 were randomly divided into training and internal validation cohorts, while patients from center 2 formed external validation cohort. CD34-immunohistochemistry was used as the reference standard for MVPs to classify patients into non-angiogenic alveolar (NAA) and non-NAA groups. Radiomics and pathomics features were extracted to construct single-phase radiomics, combined radiomics, and pathomics models. Rad-score and Path-score were derived from combined radiomics and pathomics models, respectively. Rad-score, Path-score, and clinicopathological independent predictors were integrated to develop a nomogram. Model performance was assessed by area under the curve (AUC), calibration curve, decision curve analysis (DCA), and DeLong test.

**Results:**

On multivariable analysis, histological grade was an independent predictor of NAA MVP. Combined radiomics model for predicting MVPs achieved AUCs of 0.863, 0.856, and 0.849 in training, internal validation, and external validation cohorts, showing better performance than single-phase models. Pathomics model yielded AUCs of 0.878, 0.860, and 0.833, however, its specificity markedly decreased in validation cohorts. Nomogram model achieved the superior performance across all cohorts, with AUCs of 0.911, 0.903, and 0.901, outperforming single-modality models (DeLong test: all *p* < 0.05).

**Conclusion:**

The nomogram demonstrated high accuracy and robustness in predicting MVPs in NSCLC, offering a promising tool for characterizing the tumor microenvironment and supporting individualized treatment.

## Key Messages

- NSCLC displays heterogeneous microvascular patterns (MVPs), including a non-angiogenic alveolar (NAA) subtype that co-opts pre-existing alveolar vasculature, conferring high invasiveness and resistance to anti-angiogenic therapy.
- Compared to pathomics, the combined radiomics model from preoperative multiphase CT demonstrates considerable accuracy, positioning it as a first-line and non-invasive tool for predicting MVPs in NSCLC.
- The integrated nomogram outperformed single-modality models, demonstrating the potential of radiomics-based multi-omics models for characterizing the tumor microenvironment.

## Introduction

Non-small cell lung cancer (NSCLC) is the leading cause of cancer-related deaths worldwide, representing a substantial public health challenge [1,2]. Previous studies have demonstrated that angiogenesis, characterized by pathological neovascularization, critically regulates tumor invasion, metastasis and prognosis in NSCLC [3**–**5]. Therefore, anti-angiogenic therapy, either as monotherapy or in combination regimens, has become an essential component of the treatment strategies for NSCLC [6,7]. However, clinical evidence reveals limited therapeutic efficacy in certain patients, showing significant inter-individual heterogeneity.

Pezzella et al. discovered that in highly vascularized organs (e.g. lung, brain, liver, and kidney), tumors can partially or even fully sustain progression by vascular co-option (VCo), without new vessels formation [8]. The classical cancer hallmark of ‘inducing angiogenesis’ has recently been redefined as ‘inducing and accessing vasculature’ [9]. This refinement highlighted the heterogeneity of tumor microvascular patterns (MVPs), and unveiled a new field of cancer biology research. Recent studies have implicated the non-angiogenic phenotype and its dynamic transition in response to therapeutic intervention as a potential mechanism for resistance to anti-angiogenic therapy [10–12].

NSCLC exhibited non-angiogenic alveolar (NAA) MVP in 10–40% of cases, marked by co-opted pre-existing alveolar capillary beds [13,14]. Compared to non-NAA MVPs, NAA MVP tumors presented more aggressive and metastatic behavior through effective mitochondrial metabolism and increased motility [15–17]. Risk scores based on non-angiogenic gene profiling showed that lung adenocarcinoma (LUAD) patients with high scores exhibited an immunosuppressive microenvironment and poorer outcomes, whereas low scores subgroup displayed enhanced immunotherapy response and durable clinical benefits [18]. Lin et al. demonstrated that a novel anti-angiogenic agent dually targeting both angiogenic and non-angiogenic pathways in NSCLC achieved superior antitumor efficacy *in vitro* and *in vivo* [19]. Clinical data further supported combined anti-angiogenics and anti-VCo strategies as emerging approaches for NAA tumors, with epithelial–mesenchymal transition identified as a new therapeutic target [20–22]. Therefore, NAA MVP can serve as a valuable biomarker for optimizing treatment strategies and improving prognosis in NSCLC.

Currently, the determination of tumor MVPs relies on CD34-immunohistochemical (IHC) staining. However, this method requires an invasive biopsy and may fail to fully capture tumor heterogeneity due to sampling bias. Thus, it is highly desirable to develop efficient and dynamic approaches for evaluating tumor MVPs to facilitate precision medicine.

Radiomics, through high-throughput image analysis, offers a potential non-invasive solution for personalized medicine. Multiple studies have demonstrated that CT-based radiomics models exhibit strong predictive performance for pathological features, driver gene mutations, treatment response, and survival outcomes in NSCLC [23**–**26]. Meanwhile, whole-slide image (WSI)-based pathomics enables the capture of revealing sub-visual tumor features. Yi et al. introduced a fully convolutional neural network approach for automated microvessel detection in H&E-stained images, providing a novel insight for microvascular quantification [27]. The multi-omics model integration of macroscale radiological data and microscale morphological information has redefined ‘digital biopsy’, significantly enhancing comprehensive characterization and prognostic prediction for heterogeneous tumors [28,29]. Notably, the application of multi-omics approaches for predicting MVPs in NSCLC remains limited.

Therefore, in this study we aimed to develop and validate machine learning models based on preoperative multiphase CT images and H&E-stained WSIs to predict MVPs in NSCLC.

## Materials and methods

### Patient

This study was conducted in accordance with the Declaration of Helsinki and was approved by the Institutional Review Board of Shanxi Bethune Hospital (center 1) and The People’s Hospital of Longhua (center 2) (YXLL-2025-129 and 2024011, respectively). The requirement for informed consent was waived due to the retrospective design and the use of de-identified data. Patients were consecutively screened at center 1 between October 2021 and December 2024 and at center 2 between January 2023 and December 2024, according to predefined inclusion and exclusion criteria. A total of 258 patients were included across the two medical centers. Data analysis was initiated in June 2025. Patients from center 1 were randomly allocated into the training cohort (*n* = 132) and the internal validation cohort (*n* = 57) at a 7:3 ratio. Patients from center 2 (*n* = 69) were included in the external validation cohort. All included patients underwent preoperative multiphase CT and postoperative histopathological examinations including hematoxylin-eosin (H&E) staining and CD34-IHC analysis.

**Figure 1.**
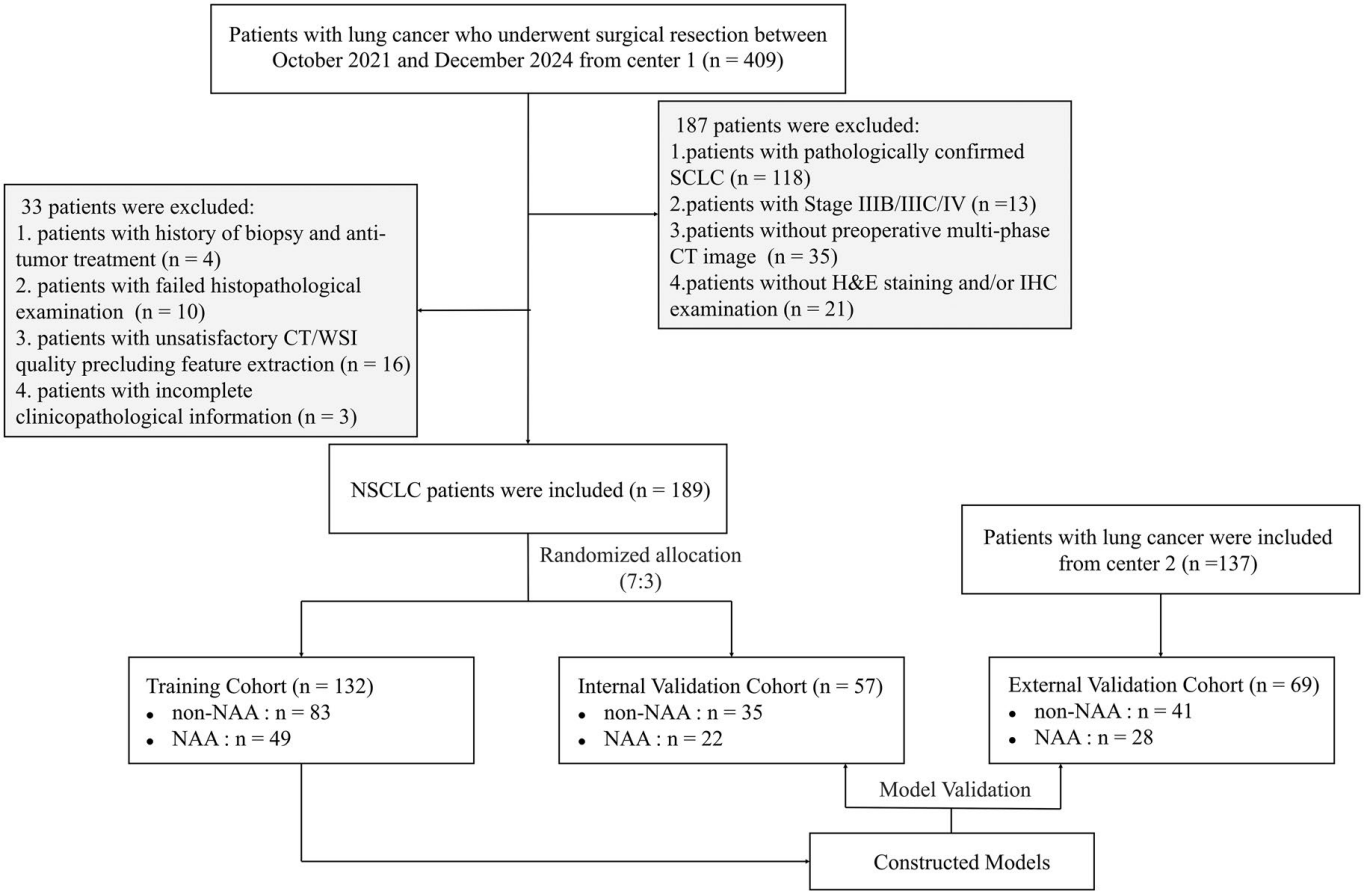
Flowchart of the patient selection process.

Inclusion criteria: (1) pathologically confirmed NSCLC with stage I-IIIA; (2) underwent unenhanced CT (UECT) and dual-phase contrast-enhanced CT (CECT), including CECT-arterial phase (CECT-AP) and CECT-venous phase (CECT-VP), within 2 weeks before surgery; (3) underwent postoperative H&E-staining and CD34-IHC examinations. Exclusion criteria: (1) history of biopsy or antitumor treatment before CT scan; (2) incomplete clinicopathological or imaging data; (3) failed IHC examination preventing MVPs evaluation; and (4) poor-quality CT (e.g. artifacts) or WSI (e.g. out-of-focus regions), resulting in failed tumor segmentation or radiomics/pathomics feature extraction. The patient inclusion flowchart is illustrated in Figure 1.

### CT image acquisition

All patients underwent multiphase chest CT examinations using one of three multidetector CT scanners (SOMATOM Definition Flash and SOMATOM Definition AS, Siemens Medical Solutions, Forchheim, Germany; GE Revolution 256 detector CT, GE Healthcare, USA). The scanning protocols included UECT, CECT-AP, and CECT-VP. The contrast agent iodixanol (320 mgI/mL) was injected at 1.5 mL/kg (3.0 mL/s) *via* an antecubital vein followed by 20 mL of saline. Using aortic arch bolus tracking (threshold 100 HU), AP scans started 7 s post-trigger, with VP acquired 30 s after AP. Reconstructed images were generated using scanner-specific slice thickness and reconstruction kernels, as detailed in Table 1, and radiomics analyses were performed on these thin-slice reconstructions.

**Table 1.** The specific parameters of three CT scanners.

CT scanner	Detector collomation	Tube voltage	Tube current	Rotation time	Pitch	Image matrix	Slice thickness	Convolution kernel
SOMATOM Definition Flash	128 × 0.6 mm	120 kV	Auto	0.50 s	1.0	512 × 512	1 mm	B70f
SOMATOM Definition AS	64 × 0.6 mm	120 kV	Auto	0.50 s	1.0	512 × 512	1 mm	B70f
GE Revolution 256 detector CT	128 × 0.625 mm	120 kV	Auto	0.50 s	1.375	512 × 512	0.625 mm	Lung

### Histopathological examination and whole slide images (WSIs) acquisition

NSCLC specimens were fixed in 10% formalin, paraffin-embedded, and sectioned at 4 μm thickness for H&E staining and CD34-IHC. Then, all sections were scanned using a scanner (Pannoramic MIDI, 3DHISTECH, Hungary) to generate WSIs.

Two experienced pathologists evaluated CD34-IHC stained sections to determine tumor MVPs in NSCLC using a consensus approach. MVPs were defined as angiogenic (including basal (BA), diffuse (DA), and papillary (PA)) and non-angiogenic alveolar (NAA) subtypes [30]. The proportion of four MVPs within CD34-IHC WSIs was estimated in 5% increments. Patients were divided into the non-NAA group and the NAA group based on the presence of NAA MVP covering ≥5% of the tumor area [14]. Figure S1 shows representative CD34-IHC sections of the four MVPs.

### CT image segmentation and feature extraction

All acquired multiphase CT images were archived in DICOM format. Two radiologists with 10 and 12 years of thoracic imaging experience, respectively, independently performed manual slice-by-slice segmentation of volumes of interest (VOIs) using ITK-SNAP software (version 4.0.1, http://www.itksnap.org). The radiologists were blinded to MVPs results. The segmented images underwent *z* score normalization to mitigate grayscale variations across different CT scan devices and batches. Subsequently, the images were resampled to standardize their spatial resolution to 1 mm × 1mm × 1mm, employing linear interpolation to adjust voxel spacing while preserving structural information and ensuring spatial consistency. Radiomics features were extracted from the UECT, CECT-AP, and CECT-VP images for each tumor VOI.

To ensure segmentation and feature extraction reproducibility, we assessed intra- and inter-observer agreement using intraclass correlation coefficients (ICCs). Thirty cases randomly selected were used to calculate inter-observer ICCs between the two radiologists. Subsequently, the same radiologist re-annotated these cases after 1 month to determine intra-observer reproducibility. Features with ICCs >0.80 were retained for further analysis. For each tumor VOI, 1306 radiomic features (RFs) were extracted using the Python PyRadiomics package (version 3.0.1), including 9 shape features, 18 first-order features, and 1,279 textural features.

### Whole slide images (WSIs) segmentation and feature extraction

Two pathologists with 12 and 16 years of experience, respectively, who were blinded to the CT imaging and clinical information, determined tumor boundaries and manually segmented tumor regions on H&E-stained WSIs using QuPath software through a consensus approach. The WSIs were scanned at ×40 magnification and stored in SVS format with a pixel resolution of 0.25 μm/pixel. During annotation, the following areas were excluded: necrotic regions (nuclear fragmentation index >30%), hemorrhagic regions, folded regions, and blurred regions (Sobel gradient <50).

To mitigate staining-related batch effects and inter-instrument variability, WSIs were color-normalized using the Macenko method. First, optical density transformation was applied to convert RGB images into the optical density space to separate stain-specific signals. Second, principal component vectors of H&E-stained slides were extracted using singular value decomposition. Third, stain vectors were mapped to a laboratory standard color palette to achieve cross-sample staining consistency. Furthermore, *z* score normalization was applied to image intensity to reduce brightness variations and normalize data distribution. Pathomics features extraction were performed using CellProfiler (version 4.2.6, https://CellProfiler.org). The H&E staining images were separated into hematoxylin and eosin grayscale images using the ‘UnmixColors’ module. Images noise were reduced *via* the ‘Smooth’ module. Nuclear and cytoplasm segmentation were automatically performed on the hematoxylin and eosin grayscale images using the ‘IdentifyPrimaryObjects’ and ‘IdentifySecondaryObjects’ modules, respectively. Cellular and subcellular pathomics features were extracted using ‘MeasureObjectSizeShape’, ‘MeasureObjectIntensity’, ‘MeasureTexture’, ‘MeasureGranularity’, and ‘MeasureObjectIntensityDistribution’ modules. For each case, all pathology features were aggregated at the patient level by calculating mean, median, standard deviation, 25th and 75th percentiles, ultimately generating a comprehensive set of 1,270 pathomics features, including 260 morphological features, 320 intensity-based features, 430 textural features, and 260 topological features.

### Radiomics and pathomics features selection

To ensure the selected radiomics and pathomics features were significant and independent, all feature selection procedures were performed exclusively within the training cohort, whereas the internal and external validation cohorts were used only for model evaluation. First, Pearson correlation analysis was employed to eliminate features with a correlation coefficient >0.90. Subsequently, univariate analysis was applied to retain features with *p* < 0.05 as a preliminary dimensionality-reduction step. Then, univariate logistic regression was used to assess the predictive capability of selected features. Finally, a five-fold cross-validation combined with the least absolute shrinkage and selection operator (LASSO) algorithm was utilized to further screen the significant features.

### Models construction and validation

**Figure 2.**
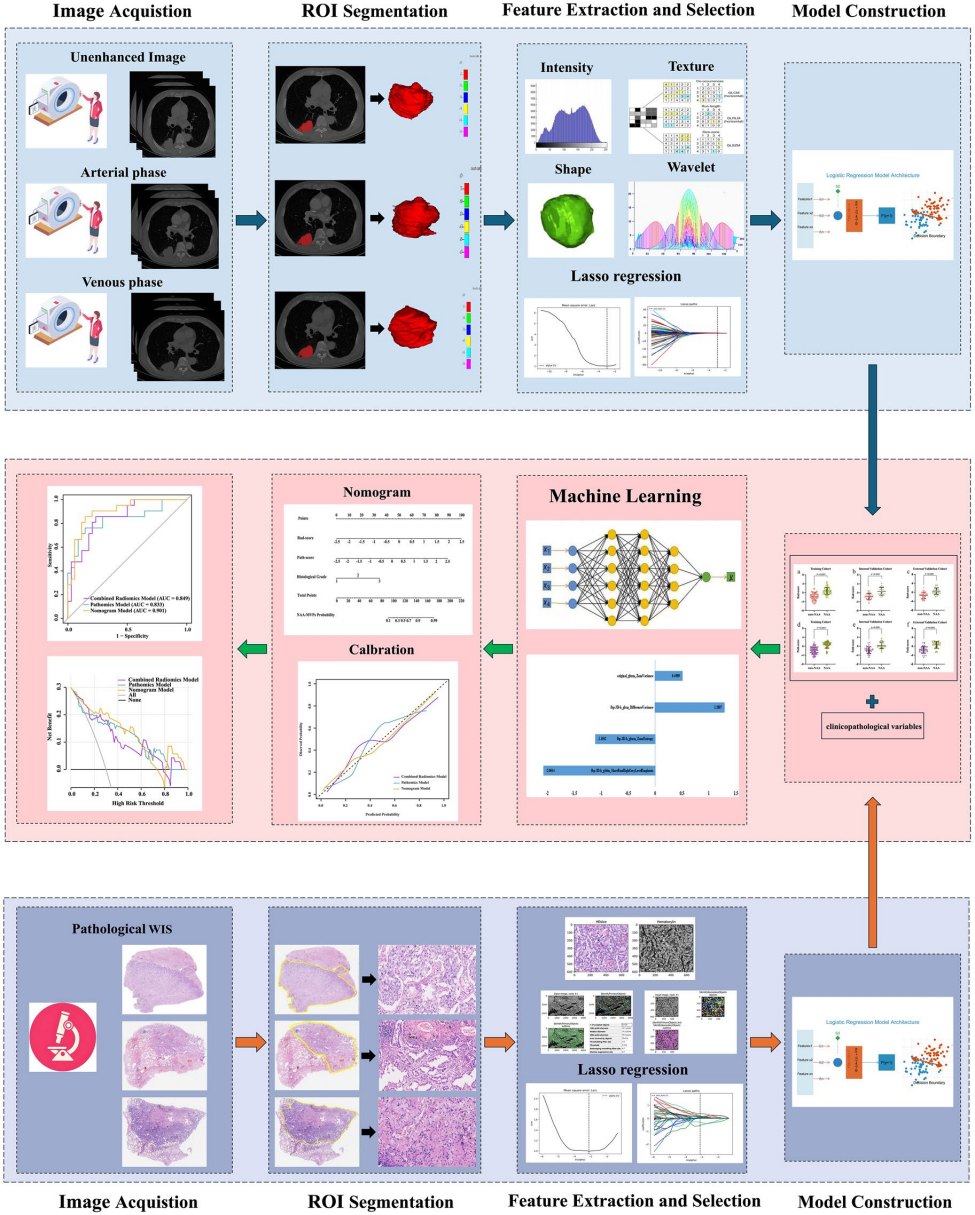
Flowchart of the study design. A series of radiomics and pathomics models were constructed using preoperative multiphase CT images and H&E-stained slides of NSCLC. Finally, radiomics-score, pathomics-scores, and clinicopathological variables were integrated to develop a multi-omics nomogram for predicting angiogenenic microvascular patterns of NSCLC.

The study design flowchart is presented in Figure 2. Based on the optimal features, we developed one pathomics model and four radiomics models (including UE model, AP model, VP model and combined radiomics model) using logistic regression to predict tumor MVPs in NSCLC.

A radiomics score (Rad-score) and a pathomics score (Path-score) were calculated through separate linear combinations of combined radiomics features and pathomics features, respectively. Univariate and multivariate logistic regression were used to select valuable clinicopathological parameters. Finally, the nomogram integrated clinicopathological variables, Rad-score and Path-score was constructed to predict tumor MVPs in NSCLC.

The predictive performance of the radiomics models, pathomics model, and nomogram were evaluated using receiver operating characteristic (ROC) analysis with area under the curve (AUC), accuracy (Acc), sensitivity (Sen), specificity (Spe), positive predictive value (PPV), and negative predictive value (NPV) in internal and external validation cohorts. DeLong’s test was performed to compare AUC values of the different prediction models. Net reclassification improvement (NRI) and integrated discrimination improvement (IDI) were calculated to quantify the incremental predictive value of the nomogram compared with the single-modality models. Calibration curves, calibration slopes, Brier scores, and the Hosmer–Lemeshow test were used to evaluate the agreement between predicted and observed probabilities. Decision curve analysis (DCA) was used to assess the clinical utility of the prediction models. Shapley additive explanation (SHAP) was performed to quantify the contribution of each feature to the model’s predictions and to improve model interpretability.

Overall, the study design and reporting followed the core methodological principles of the radiomics quality score (RQS), applied to both radiomics and pathomics analyses.

### Statistical analysis

Statistical analyses were performed using SPSS v22.0 and R software (version 4.1.2, http://www.Rproject.org). Continuous variables were expressed as mean ± standard deviation (normally distributed) or median (interquartile range), while categorical variables were presented as frequencies and percentages. Independent samples *t* tests were used for normally distributed data, and Mann–Whitney *U* tests were applied for non-normally distributed data. Categorical variables were compared using chi-square tests or Fisher’s exact test. A two-sided *p* value <0.05 was considered statistically significant.

## Results

### Clinicopathological characteristics

Finally, 258 patients with NSCLC were included (center 1: *n* = 189; center 2: *n* = 69), with a mean age of 60.37 ± 10.09 years. There were 141 males (54.7%) and 117 females (45.3%), including 197 cases of LUAD, 56 cases of squamous cell carcinoma (SCC), and 5 cases of other rare subtypes. 176 tumors (68.2%, 176/258) exhibited two or more MVPs. Using the predefined NAA coverage threshold of ≥5% of the tumor area, 258 patients were divided into the NAA group (*n* = 99, 38.4%) and non-NAA group (*n* = 159, 61.6%). In the training, internal validation and external validation cohorts, the number of patients in the NAA group was 49 (37.1%, 49/132), 22 (38.6%, 22/57), and 28 (40.6%, 28/69). Table 2 summarized the clinicopathological characteristics of NSCLC patients across three cohorts. Histological grade and type showed significant differences between NAA and non-NAA groups across the three cohorts (*p* < 0.05), with microvessel area (MVA) demonstrating significant variations in the training and external validation cohorts. No significant differences were observed for other clinicopathological factors. In the training cohort, univariate and multivariate analyses of 12 clinicopathological factors identified histological grade (OR = 2.671; 95% CI, 1.499–4.759; *p* < 0.001) as an independent predictor of NAA MVP (Table 3).

**Table 2.** Clinicopathological characteristics of NSCLC patients in the training, internal validation and external validation cohorts.

Characteristics	Training cohort (*n* = 132)			Internal validation cohort (*n* = 57)			External validation cohort (*n* = 69)		
	NAA group (*n* = 49)	Non-NAA group (*n* = 83)	*p*	NAA group (*n* = 22)	Non-NAA group (*n* = 35)	*p*	NAA group (*n* = 28)	Non-NAA group (*n* = 41)	*p*
Age (years), (mean ± SD)	60.13 ± 10.71	60.03 ± 8.14	0.952	61.47 ± 10.96	61.52 ± 10.45	0.986	60.11 ± 13.58	59.98 ± 9.19	0.962
Gender, *n* (%)			0.183			0.927			0.430
Male	26 (53.1%)	48 (57.8%)		10 (45.5%)	19 (54.3%)		15 (53.6%)	23 (56.1%)	
Female	23 (46.9%)	35 (42.2%)		12 (54.5%)	16 (45.7%)		13 (46.4%)	18 (43.9%)	
Smoking, *n* (%)			0.940			0.226			0.496
No	29 (59.2%)	49 (59.0%)		8 (36.4%)	17 (48.6%)		18 (64.3%)	23 (56.1%)	
Yes	20 (40.8%)	34 (41.0%)		14 (63.6%)	18 (51.4%)		10 (35.7%)	18 (43.9%)	
Histological type, *n* (%)			<0.001*			0.003*			0.021*
Other	1 (2.0%)	1 (1.2%)		0 (0.0%)	1 (2.9%)		0 (0.0%)	2 (4.9%)	
LUAD	47 (96.0%)	54 (65.1%)		22 (100%)	19 (54.3%)		28 (100%)	27 (65.9%)	
LUSC	1 (2.0%)	28 (33.7%)		0 (0.0%)	15 (42.8%)		0 (0.0%)	12 (29.2%)	
Histological grade, *n* (%)			0.011*			0.016*			0.032*
1(well)	12 (24.5%)	41 (49.4%)		6 (27.3%)	12 (34.3%)		3 (10.7%)	16 (39.0%)	
2(moderate)	34 (69.4%)	36 (43.4%)		12 (54.5%)	23 (65.7%)		22 (78.6%)	23 (56.1%)	
3(poor)	3 (6.1%)	6 (7.2%)		4 (18.2%)	0 (0.0%)		3 (10.7%)	2 (4.9%)	
T stage, *n* (%)			0.172			0.394			0.132
T1	30 (61.2%)	46 (55.4%)		15 (68.2%)	24 (68.5%)		19 (67.9%)	24 (58.5%)	
T2	16 (32.7%)	23 (27.7%)		3 (13.6%)	3 (8.6%)		6 (21.4%)	9 (22.0%)	
T3	1 (2.0%)	11 (13.3%)		4 (18.2%)	5 (14.3%)		2 (7.1%)	6 (14.6%)	
T4	2 (4.1%)	3 (3.6%)		0	3 (8.6%)		1 (3.6%)	2 (4.9%)	
*N* stage, *n* (%)			0.291			0.874			0.167
*N*0	42 (85.7%)	62 (74.7%)		19 (86.4%)	31 (88.6%)		26 (92.9%)	35 (85.4%)	
*N*1 + 2	7 (14.3%)	21 (25.3%)		3 (13.6%)	4 (11.4%)		2 (7.1%)	6 (14.6%)	
TNM stage, *n* (%)			0.308			0.886			0.107
I	37 (75.5%)	54 (65.1%)		15 (68.2%)	24 (68.5%)		22 (78.6%)	26 (63.4%)	
II	7 (14.3%)	15 (18.1%)		4 (18.2%)	6 (17.2%)		3 (10.7%)	7 (17.1%)	
III	5 (10.2%)	14 (16.8%)		3 (13.6%)	5 (14.3%)		3 (10.7%)	8 (19.5%)	
LVI, *n* (%)			0.352			0.962			0.729
No	41 (83.7%)	67 (80.7%)		18 (81.8%)	30 (85.7%)		25 (89.3%)	34 (82.9%)	
Yes	8 (16.3%)	16 (19.3%)		4 (18.2%)	5 (14.3%)		3 (10.7%)	7 (17.1%)	
PI, *n* (%)			0.501			0.977			0.706
No	43 (87.8%)	74 (89.2%)		21 (95.5%)	32 (91.4%)		24 (85.7%)	37 (90.2%)	
Yes	6 (12.2%)	9 (10.8%)		1 (4.5%)	3 (8.6%)		4 (14.3%)	4 (9.8%)	
TLD (cm)	2.83 ± 1.47	3.30 ± 2.26	0.162	2.79 ± 2.19	3.08 ± 2.53	0.663	2.68 ± 1.91	3.37 ± 2.77	0.254
CD34-MVD, Median (IQR)									
MVC	32.33 (32.00)	29.17 (38.25)	0.856	42.00 (39.01)	29.83 (24.08)	0.249	36.17 (31.08)	36.67 (32.17)	0.562
MVA	1.11 (1.33)	1.64 (2.08)	0.003*	2.03 (1.72)	1.38 (2.64)	0.628	1.06 (1.19)	2.21 (2.77)	0.003*

*Note. p* < 0.05 and ‘*’ was considered to have a statistical difference.

CD34-MVD CD34-immunohistochemical assessed microvessel density, MVC microvessel count, MVA microvessel area, NAA non-angiogenic alveolar, LUAD lung adenocarcinoma, LUSC lung squamous cell carcinoma, LVI lymphovascular invasion, PI Pleural invasion, TLD tumor long diameter.

**Table 3.** Univariate and multivariate analyses of clinical characteristics between NAA and non-NAA subgroups in the training cohort.

Variables	Univariate analysis			Multivariate analysis		
	β	OR(95%CI)	*p* value	β	OR(95%CI)	*p* value
Gender	−0.494	0.610 (0.332–1.120)	0.111			
Age	0.001	1.000 (0.970–1.033)	0.966			
Smoking	−0.192	0.825 (0.440–1.528)	0.541			
Histological type						
Other			0.866			
LUAD	20.510	0.236 (0.158–0.353)	0.997			
LUSC	21.173	0.264 (0.171–0.406)	0.997			
Histological grade	0.711	2.036 (1.200–3.453)	0.008*	0.982	2.671(1.499–4.759)	<0.001*
T stage	−0.220	0.248 (0.553–1.165)	0.248			
N stage	−0.283	0.754 (0.468–1.215)	0.246			
TNM stage	−0.302	0.739 (0.485–1.128)	0.161			
LVI	−0.227	0.797 (0.336–1.890)	0.606			
PI	0.157	1.170 (0.423 ∼ 3.235)	0.762			
TLD	−0.099	0.906 (0.777–1.056)	0.207			
CD34-MVD						
MVC	−0.002	0.998 (0.988–1.008)	0.728			
MVA	−0.174	0.841 (0.709–0.996)	0.045*	−0.131	0.877(0.738–1.042)	0.135

CD34-MVD CD34-immunohistochemical assessed microvessel density, MVC microvessel count, MVA microvessel area, LUAD lung adenocarcinoma, LUSC lung squamous cell carcinoma, LVI lymphovascular invasion, PI Pleural invasion, TLD, tumor long diameter.

### Construction and evaluation of four radiomics models

Following Pearson correlation analysis, univariate correlation analysis, univariate logistic regression, and the LASSO algorithm, 5, 6, 6, and 8 optimal RFs were selected from 1306 RFs to construct the UE, AP, VP, and combined radiomics models, respectively (Figure S2, detailed features in Table S1). The selected radiomic features and their corresponding coefficients for the combined radiomics model are presented in Figure S3.

**Figure 3.**
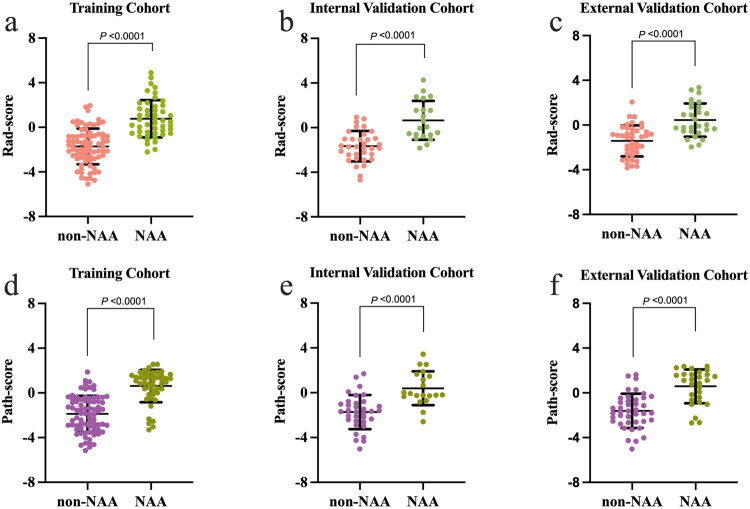
Violin plots illustrating the distribution of Rad-scores (**a–c**) and Path-scores (**d–f**) between non-NAA and NAA subgroups in the training, internal validation, and external validation cohorts of NSCLC patients. Across all cohorts, the Rad-score and Path-score were significantly higher in the NAA group compared to the non-NAA group.

As shown in Table 4 and Figure S4, among single-phase radiomics models, the VP model demonstrated the highest predictive performance for NAA MVP compared to UE and AP models (DeLong test: *p* < 0.05; Table S2 in the Supplement Materials), achieving AUCs of 0.841 (95% CI: 0.780–0.849), 0.831 (95% CI: 0.727–0.840), and 0.835 (95% CI: 0.735–0.843) in training, internal validation, and external validation cohorts, respectively. Combined radiomics model based on multiphase CT significantly outperformed single-phase models, with AUC improving to 0.863 (95% CI: 0.851–0.870), 0.856 (95% CI: 0.848–0.869), and 0.849 (95% CI: 0.837–0.852) across the three cohorts, respectively (DeLong test: *p* < 0.05; Table S2). Calibration curves for four radiomics models are depicted in Figure S5. Hosmer–Lemeshow tests confirmed excellent agreement between predicted and actual microvascular patterns in NSCLC across cohorts (all *p* > 0.05, Table S2).

**Figure 4.**
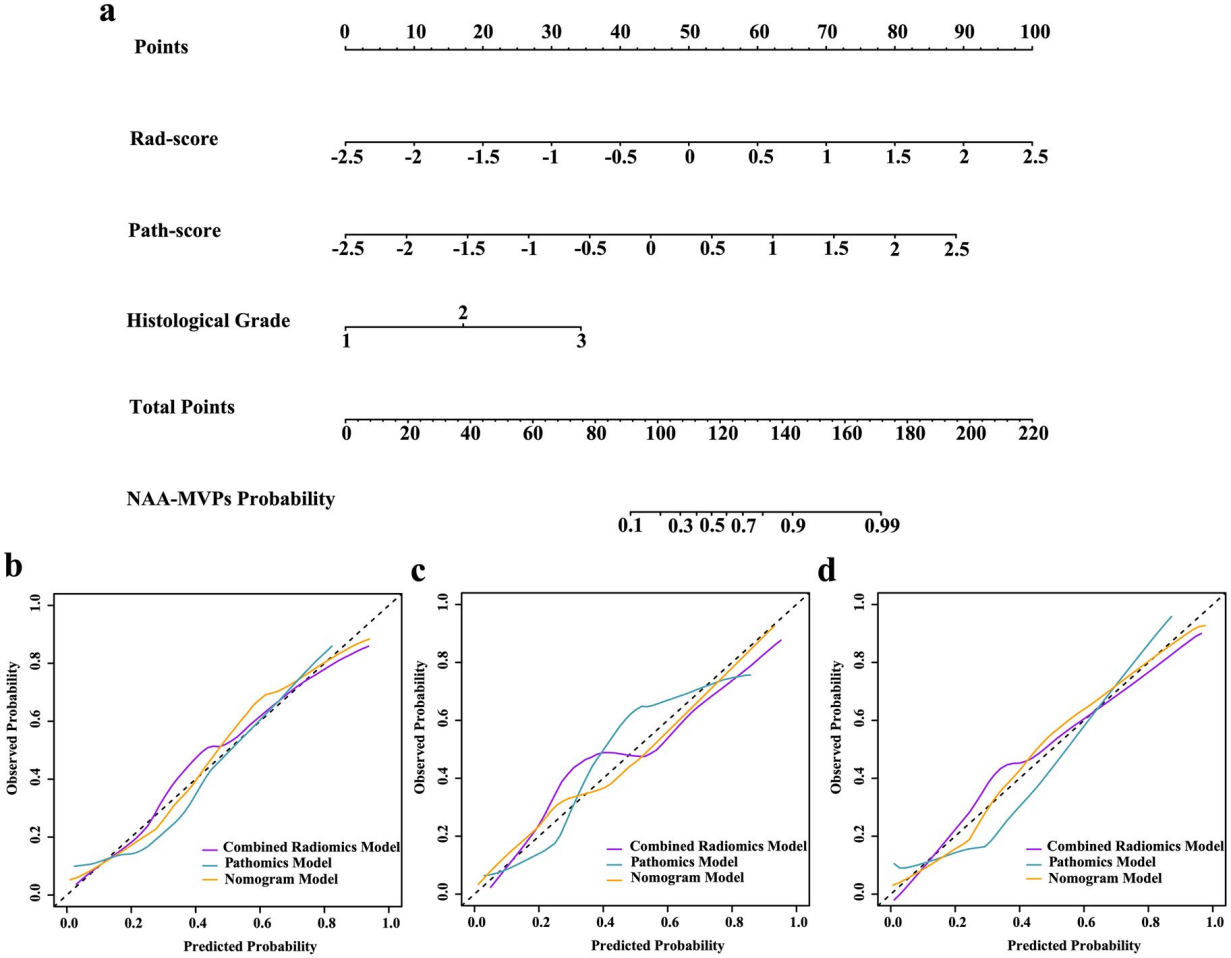
Construction and calibration of the integrated nomogram model. (**a**) The nomogram incorporating Rad-score, Path-score, and histological grade for predicting NAA–MVPs in NSCLC patients. (**b–d**) Calibration plots of the combined radiomics, pathomics, and nomogram models in the training cohort (**b**), internal validation cohort (**c**), and external validation cohort (**d**).

**Figure 5.**
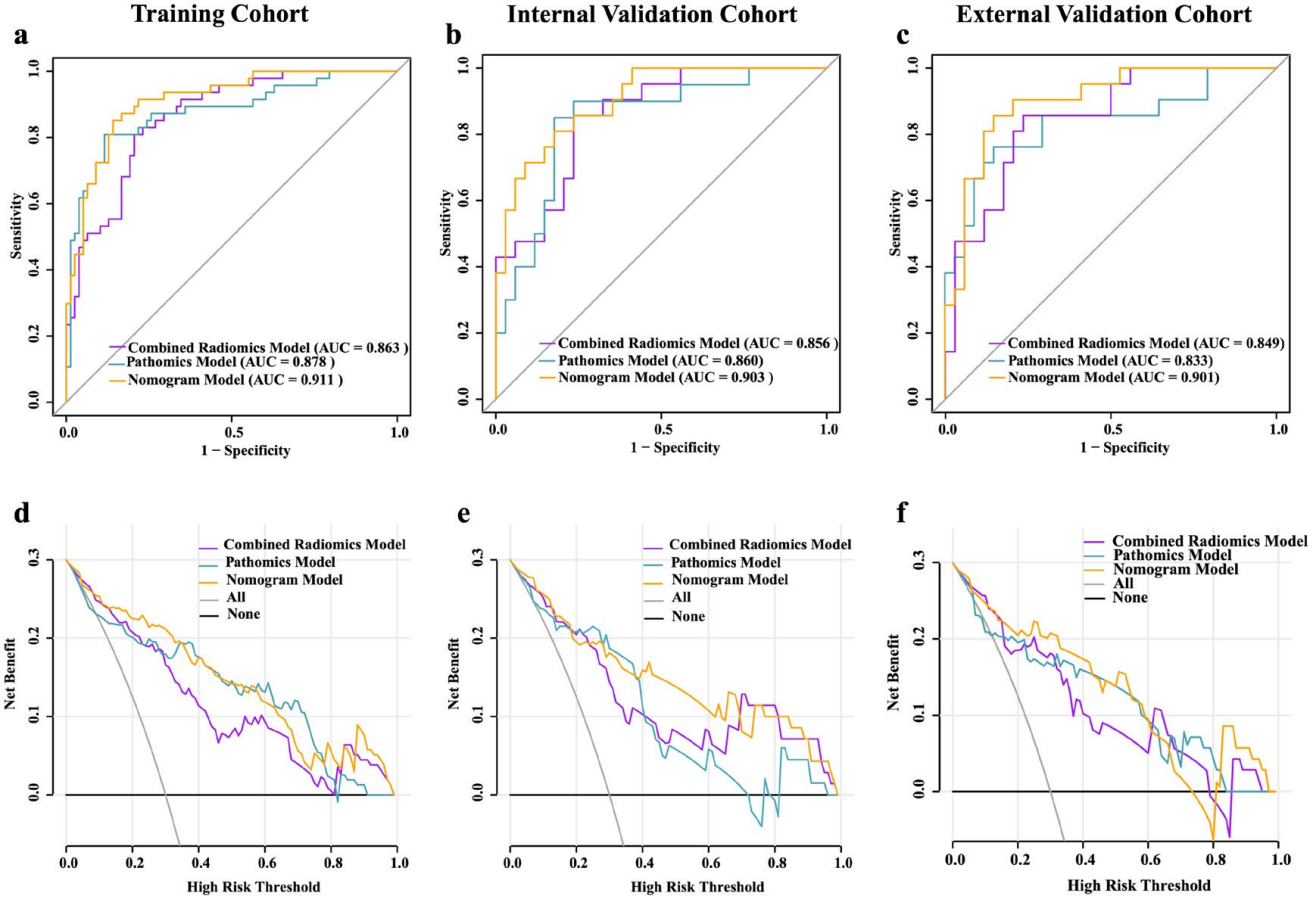
Predictive performance of different models in this study. (**a–c**) ROC curves of combined radiomics, pathomics and nomogram models in the training cohort (**a**), internal validation cohort (**b**), and external validation cohort (**c**). (**d–f**) DCA of combined radiomics, pathomics and nomogram models in the training cohort (**d**), internal validation cohort (**e**), and external validation cohort (**f**); ROC receiver operating characteristic, DCA decision curve analysis.

**Table 4.** Comparison of the predictive performance of four Radiomics models, Pathomics model and radiopathomics Nomogram model for NAA MVP in NSCLC.

	AUC (95% CI)	ACC	Sens	Spec	PPV	NPV
**Training cohort**						
UE radiomics	0.812 (0.746–0.825)	0.712	0.657	0.628	0.615	0.861
AP radiomics	0.833 (0.773–0.848)	0.744	0.683	0.697	0.580	0.873
VP radiomics	0.841 (0.780–0.849)	0.760	0.701	0.705	0.635	0.875
Combined radiomics	0.863 (0.851–0.870)	0.792	0.805	0.795	0.698	0.883
Pathomics	0.878 (0.875–0.884)	0.848	0.784	0.885	0.780	0.887
Nomogram	0.911 (0.898–0.952)	0.848	0.830	0.859	0.804	0.893
**Internal validation cohort**						
UE radiomics	0.807 (0.701–0.827)	0.679	0.667	0.588	0.562	0.605
AP radiomics	0.822 (0.725–0.831)	0.683	0.651	0.559	0.559	0.670
VP radiomics	0.831 (0.727–0.840)	0.691	0.714	0.647	0.556	0.750
Combined radiomics	0.856 (0.848–0.869)	0.745	0.851	0.794	0.667	0.786
Pathomics	0.860 (0.865–0.884)	0.759	0.624	0.836	0.727	0.794
Nomogram	0.903 (0.895–0.961)	0.800	0.851	0.824	0.786	0.848
**External validation cohort**						
UE radiomics	0.812 (0.706–0.827)	0.691	0.683	0.618	0.567	0.640
AP radiomics	0.825 (0.729–0.836)	0.727	0.657	0.647	0.600	0.680
VP radiomics	0.835 (0.735–0.843)	0.745	0.782	0.794	0.667	0.794
Combined radiomics	0.849 (0.837–0.852)	0.753	0.857	0.765	0.692	0.879
Pathomics	0.833 (0.745–0.846)	0.738	0.663	0.788	0.696	0.839
Nomogram	0.901 (0.877–0.961)	0.834	0.862	0.835	0.790	0.857

CI confidence intervals, AUC area under curve, ACC accuracy; Sens, sensitivity, Spec specificity, PPV positive predictive value, NPV negative predictive value.

### Construction and evaluation of the pathomics model

After rigorous feature selection utilizing Pearson correlation analysis and LASSO algorithm, 3 optimal features were selected from 1270 pathomics features for model construction (Figure S6, detailed features in Table S3).

**Figure 6.**
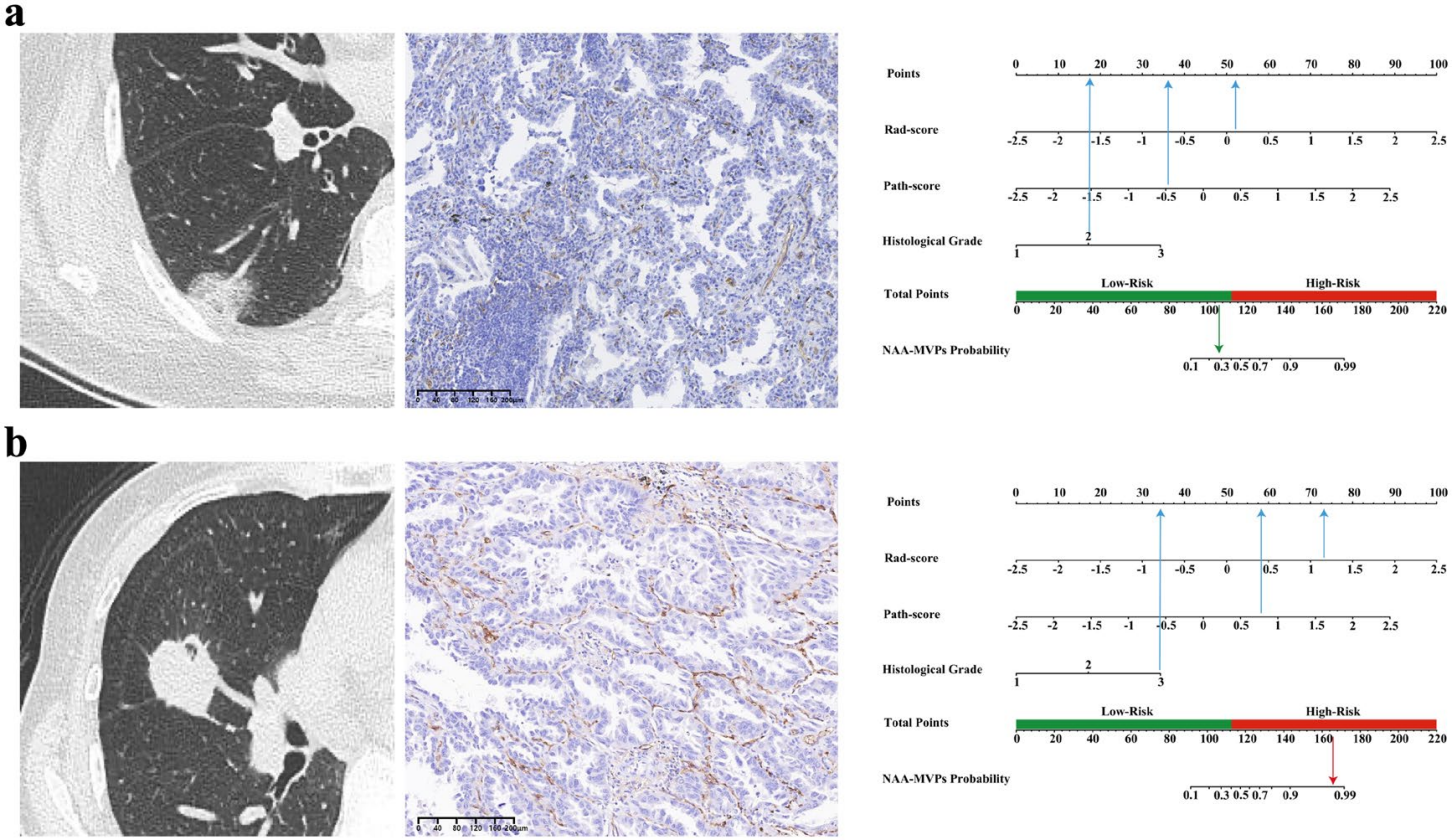
Clinical application of the nomogram for predicting NAA–MVP in two representative NSCLC cases. (**a**) Case 1: A patient with pathologically confirmed moderately differentiated lung adenocarcinoma, manifesting as a mixed ground-glass nodule in the right lower lobe on CT images. CD34-Immunohistochemical (IHC) staining (×100) revealed a papillary angiogenic (PA) microvessel pattern (MVP), confirming non-NAA–MVPs. The total points calculated from the nomogram were 105.91, corresponding to an approximately 30% probability of NAA–MVPs. (**b**) Case 2: A patient with pathologically confirmed poorly differentiated lung adenocarcinoma, manifesting as a solid nodule in the right middle lobe on CT images. CD34-IHC staining (×100) confirmed NAA–MVP. The total points derived from the nomogram were 165.27, corresponding to a greater than 95% probability of NAA–MVP.

As shown in Table 4, Pathomics model achieved AUCs of 0.878 (95% CI: 0.875–0.884) and 0.860 (95% CI: 0.865–0.884) in the training and internal validation cohorts, respectively, outperforming the combined radiomics model (DeLong test: *p* = 0.009 and *p* = 0.020). However, its performance declined in the external validation cohort (AUC: 0.833 vs. 0.849; DeLong test: *p* = 0.009). Meanwhile, we observed reduced sensitivity in the Pathomics model, decreasing from 0.784 (training cohort) to 0.624 (internal validation cohort) and to 0.663 (external validation cohort).

### Construction and performance of an integrated radiopathomics nomogram model

Based on the selected features and their corresponding weighted coefficients for combined radiomics model and pathomics model, Rad-score and Path-score were calculated for each patient. The distribution of Rad-score and Path-score for non-NAA and NAA groups across three cohorts was shown in Figure 3. By combining clinicopathological predictors (histological grade), Rad-score, and Path-score, the integrated radiopathomics nomogram model was constructed through weighted linear combination (Figure 4a). The calibration curves (Figure 4b–d) indicated good agreement between the predicted and actual NAA outcomes in the training, internal validation, and external validation cohorts for the combined radiomics, pathomics, and nomogram models (Hosmer–Lemeshow test, *p* > 0.05; Table S2). Additionally, calibration slope and Brier score analyses (Table S4) indicated that the nomogram demonstrated the best calibration and the lowest Brier score compared with single-modality models.

As demonstrated in Table 4 and Figure 5a–c, the nomogram model achieved highest prediction performance across three cohorts compared to single-omics models (DeLong test: *p* < 0.05; Table S2). The AUCs reached 0.911 (95% CI: 0.898–0.952), 0.903 (95% CI: 0.895–0.961), and 0.901 (95% CI: 0.877–0.961) in the training, internal validation, and external validation cohorts respectively, representing improvements of 4.8%, 4.7%, and 5.2% over the Combined radiomics model, and 3.3%, 4.3%, and 6.8% over the pathomics model. The accuracy of nomogram model achieved 0.800 and 0.834 in the internal validation and external validation cohorts, respectively, surpassing the combined radiomics model by 5.5% and 8.1%, and the Pathomics model by 4.1% and 9.6%. Meanwhile, the nomogram model optimized the balance between sensitivity and specificity, achieving 0.830 and 0.859 in the training cohort, 0.851 and 0.824 in the internal validation cohort, and 0.862 and 0.835 in the external validation cohort. The nomogram improved the prediction of MVPs compared with the single-modality models, as confirmed by NRI and IDI values (Table S5).

DCA indicated that the nomogram model showed the greatest clinical net benefit for evaluating NAA MVP across a wide threshold probability range compared to combined radiomics model and pathomics model across three cohorts (Figure 5d–f). Table S6 details the points for each variable in the nomogram and the calculation formula. Figure 6 presents two clinical cases to demonstrate its clinical application. Concurrently, a distinct decline in DCA was observed for the Pathomics model within internal and external validation cohorts, whereas the combined radiomics model maintained highly consistent curve profiles across all three cohorts, indicating its potential for non-invasive assessment of NAA status in clinical decision-making.

### Models interpretability analysis

**Figure 7.**
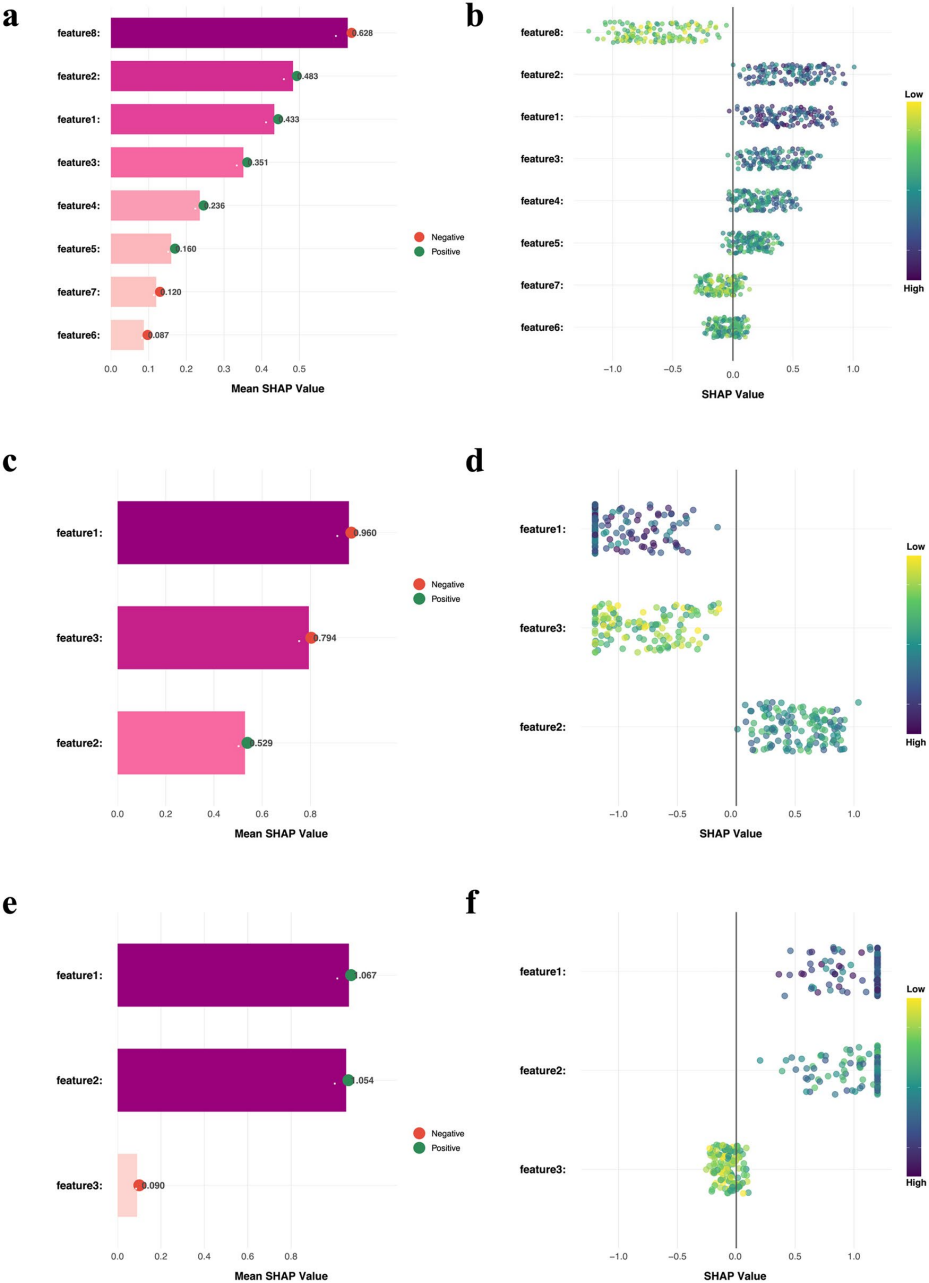
SHAP-based interpretability of the different models. (**a**,**b**) SHAP summary bar plot and beeswarm plot for the combined radiomics model. The summary bar plot ranks features by mean absolute SHAP value, indicating global importance. The beeswarm plot shows the distribution of SHAP values across samples, with each dot representing one sample. Feature1–8 correspond to: UE_wavelet_LLH_gldm_LargeDependenceEmphasis, AP_wavelet_HLH_gicm_Correlation, VP_wavelet_HLH_gicm_Correlation, UE_wavelet_LLL_gicm_InverseVariance, AP_wavelet_HLH_firstorder_Mean, VP_lbp_3D_m2_glrlm_LowGrayLevelRunEmphasis, VP_gradient_gicm_Correlation, and AP_squareroot_firstorder_Skewness. (**c,d**) SHAP summary bar plot and beeswarm plot for the Pathomics model. Feature1–3 correspond to: Texture_Entropy_Hematoxylin, AreaShape_Zernike, and Texture_InfoMeas1_Hematoxylin. (**e**,**f**) SHAP summary bar plot and beeswarm plot for the nomogram, illustrating the relative contributions of the integrated predictors (Feature 1: Rad-score; Feature 2: Path-score; Feature 3: histological grade) to the model output.

Given the complexity of the model structure, we performed SHAP to assess model interpretability by attributing the model’s predictions to individual input features. Feature contributions to the predictions were visualized and quantified using the SHAP summary bar plot and beeswarm plot, thereby facilitating a better understanding of the model’s decision-making process. As shown in Figure 7a,b, the global importance ranking indicated that the combined radiomics model was driven primarily by the following radiomics features of AP_squareroot_firstorder_Skewness, AP_wavelet_HLH_glcm_Correlation, UE_wavelet_LLH_gldm_LargeDependenceEmphasis, VP_wavelet_HLH_glcm_Correlation, UE_wavelet_LLL_glcm_InverseVariance, AP_wavelet_HLH_firstorder_Mean (mean SHAP values: 0.628, 0.483, 0.433, 0.351, 0.236, and 0.160, respectively). Figure 7c,d showed that Texture_Entropy_Hematoxylin had the largest contribution in the Pathomics model, followed by Texture_InfoMeas1_Hematoxylin and AreaShape_Zernike. In the integrated nomogram, Figure 7e–f further demonstrated that Rad-score and Path-score had similar contributions.

## Discussion

Angiogenic MVPs in primary or metastatic NSCLC were strongly associated with pluripotent stem cell expression, tumor aggressiveness and poor prognosis, while also conferring resistance to anti-angiogenic therapy [31,32]. To date, few studies have explored the prediction of NAA MVP in NSCLC using multimodal medical data. In this work, we developed radiomics models based on preoperative multiphase CT images, pathomics models derived from postoperative H&E-stained WSIs, and a nomogram model that integrated radiopathomics and clinicopathological parameters. Our findings demonstrated that the combined radiomics model based on multiphase CT achieved favorable predictive performance, highlighting its potential as a noninvasive tool for preoperative assessment. Furthermore, the nomogram model outperformed individual models across the training, internal validation, and external validation cohorts, exhibiting superior robustness and reproducibility.

Artificial intelligence (AI)-driven machine learning can quantitatively analyze high-throughput imaging features to noninvasively capture tumor phenotypes and the tumor microenvironment [33]. This ‘virtual biopsy’ approach provides a noninvasive basis for molecular profiling-guided clinical interventions and prognostic predictions in oncology. A multicenter cohort study in hepatocellular carcinoma (HCC) incorporated the MVPs characterized by vessels encapsulating tumor clusters (VETC) into MRI-based deep learning algorithms to predict post-resection recurrence-free survival (RFS), with time-dependent AUCs of 0.860, 0.800, and 0.870 at 1, 2, and 3 years, respectively [34]. Li et al. developed a nomogram model based on dual-energy CT quantitative parameters and radiomics to predict non-angiogenic MVPs in NSCLC, achieving an AUC of 0.950 in the validation cohort [35]. In our study, the VP model showed the best diagnostic accuracy among single-phase radiomics models. This finding aligned with Dong et al. who used preoperative dynamic contrast-enhanced MRI to build a deep learning radiomics model for predicting VETC-related MVPs in HCC and found that the venous-phase peritumoral model yielded the best performance [36]. Similarly, Li et al. found iodine concentration in VP derived from spectral CT was significantly correlated with angiogenesis in lung cancer [37]. We hypothesize that the superior ability of VP images to characterize angiogenic MVPs may be attributed to the increased permeability of abnormal neovasculature, leading to contrast agent retention as the distribution between vessels and tumor tissue approaches perfusion equilibrium during this phase.

Our study showed that the combined radiomics model achieved better diagnostic performance than single-phase models, highlighting the complementary value of temporal hemodynamic information. Multiphase enhancement can provide a more complete depiction of angiogenesis and tumor microenvironment. Consistent with our findings, prior studies in LUAD have shown that incorporating multiphase (or dual-phase) contrast-enhanced CT radiomics improves predictive performance over single-phase models for both molecular profiling and clinical outcomes [34,38]. We also confirmed the feasibility of Pathomics in evaluating angiogenesis using routine H&E slides, providing a potential alternative when CD34 immunohistochemistry is unavailable. Although its performance was good, specificity declined in validation cohorts, probably due to sampling bias and tumor heterogeneity. By contrast, combined radiomics model, based on non-invasive and whole-tumor assessment, avoids these limitations and may be more suitable as a first-line tool for clinical screening and patient stratification.

In this study, we developed a nomogram combining Rad-score, Path-score, and histological grade to predict NAA MVP. The model achieved higher diagnostic performance than single-omics models across all three cohorts (AUCs > 0.900, ACCs > 0.800) and demonstrated good generalizability. Accumulating evidence indicates that integrating imaging-derived features with multimodal data can improve diagnostic precision. In this context, Yao et al. developed a multimodal deep learning nomogram incorporating clinical features, multiparametric MRI, and H&E slides, demonstrating improved microsatellite instability (MSI) prediction in rectal cancer [39]. In NSCLC, Vaidya et al. developed a radiopathomics fusion model that identified high-risk patients for early recurrence and predicted response to immunotherapy and chemotherapy [40]. Although incorporating pathomics inevitably increases workflow complexity and cost, the growing clinical accessibility of WSIs and AI analytics is making radiologic-pathologic integration increasingly feasible, thereby providing comprehensive evidence for precision oncology.

Interpretability analysis and model deconstruction of data-driven features are key to improving the reliability of predictive models. In combined radiomics model, the most important radiomics features were mainly derived from first-order and wavelet-transformed features (e.g. glcm/gldm), with a squareroot intensity transformation applied to one feature. AP_squareroot_firstorder_Skewness may reflect a more ‘hot-spot’–like arterial enhancement pattern, suggesting greater early perfusion heterogeneity. AP/VP_wavelet_HLH_glcm_Correlation quantify the spatial organization of fine-scale enhancement textures after wavelet decomposition, higher values may indicate more structured and spatially correlated enhancement patterns across phases. UE_wavelet_LLH_gldm_LargeDependenceEmphasis and UE_wavelet_LLL_glcm_InverseVariance reflect baseline texture homogeneity, corresponding to larger homogeneous dependence regions and greater local uniformity, respectively. AP_wavelet_HLH_firstorder_Mean summarizes the mean intensity of the wavelet-filtered arterial-phase image, which may relate to tumor enhancement. For H&E-based pathomics, Hematoxylin-Entropy, InfoMeas1, and Zernike quantify nuclear texture heterogeneity, spatial organization, and morphologic complexity, respectively, in line with prior reports linking nuclear/cellular shape–texture features to NSCLC prognosis [41]. These features may reflect differences in tissue architecture, perfusion/permeability distribution, and invasion-related morphologic remodeling; Nevertheless, mechanistic validation linking these features to angiogenesis pathways, immune contexture, and other TME biomarkers is required in future investigations.

This study has several limitations. First, due to its retrospective design, selection bias in patient enrollment was unavoidable. Second, tumor segmentation relied on manual delineation by radiologists and pathologists, which may limit scalability and reproducibility; future work will explore AI-based automated segmentation (e.g. deep learning) to improve workflow efficiency and consistency. Finally, although patients were recruited from two centers, the overall sample size remained relatively limited, particularly in the external validation cohort. While the model showed good performance in both validation cohorts, its robustness and generalizability should be further confirmed in larger prospective multicenter cohorts and in populations beyond China.

## Conclusions

This study constructed multiple single-omics models and a nomogram by integrating clinicopathological characteristics, preoperative CT images, and H&E-stained slides to predict angiogenic MVPs in NSCLC. The results demonstrated that combined radiomics model, based on multiphase CT, showed promising potential for non-invasive preoperative evaluation. Furthermore, the fusion nomogram model achieved superior diagnostic performance and generalizability compared with individual models. The models may serve as a valuable decision-support tool to facilitate personalized treatment and improve prognosis in NSCLC patients.

## Supplementary Material

No 252689864 Supplemental Materials with clean version.docx

## Data Availability

The data generated or analyzed during this study are available from the corresponding author upon reasonable request.

## Abbreviations

AP: arterial phase
AUC: area under the curve
CECT: contrast-enhanced CT
DCA: decision curve analysis
H&E: hematoxylin-eosin
IHC: immunohistochemical
LASSO: least absolute shrinkage and selection operator
LUAD: lung adenocarcinoma
MVPs: microvascular patterns
NAA: non-angiogenic alveolar
NSCLC: non-small cell lung cancer
ROC: receiver operating characteristic
UECT: un-enhanced CT
VCo: vascular co-option
VP: venous phase
WSI: whole-slide image
