# Supplementary material for: Integrated radiopathomics nomogram for predicting angiogenic microvascular patterns in NSCLC: a dual-center validation study
Source: Ann Med. 2026 Apr 17;58(1):2654291. doi: 10.1080/07853890.2026.2654291 (PMC13094236; doi:10.1080/07853890.2026.2654291)
Supplement: No 252689864 Supplemental Materials with clean version.docx [file IANN_A_2654291_SM8763.docx]

**Integrated Radiopathomics Nomogram for Predicting Angiogenic Microvascular Patterns in NSCLC: A Dual-Center Validation Study**

**ELECTRONIC SUPPLEMENTARY MATERIALS**

**Supplementary** **Table S1.** The selected radiomics features

| Model | Feature | OR | 0.025 | 0.975 | *p* value |
| --- | --- | --- | --- | --- | --- |
| UE Model | wavelet_LLL_glcm_InverseVariance | 1.995 | 1.1 | 3.618 | 0.023 |
|  | original_firstorder_Minimum | 2.349 | 1.285 | 4.293 | 0.006 |
|  | lbp_3D_m2_firstorder_Maximum | 2.815 | 1.611 | 4.918 | 0.001 |
|  | lbp_3D_m1_glcm_Idm | 0.501 | 0.273 | 0.919 | 0.025 |
|  | wavelet_LLH_gldm_LargeDependenceEmphasis | 1.707 | 1.007 | 2.893 | 0.047 |
| AP Model | squareroot_firstorder_Skewness | 0.445 | 0.267 | 0.741 | 0.002 |
|  | wavelet_HLH_glcm_Correlation | 1.662 | 0.994 | 2.779 | 0.033 |
|  | lbp_3D_k_glszm_GrayLevelVariance | 0.551 | 0.342 | 0.889 | 0.014 |
|  | gradient_glcm_ClusterProminence | 0.578 | 0.332 | 1.005 | 0.032 |
|  | wavelet_HLH_firstorder_Mean | 0.548 | 0.345 | 0.869 | 0.011 |
|  | lbp_3D_m1_firstorder_Skewness | 1.739 | 1.065 | 2.84 | 0.027 |
| VP Moedl | lbp _3D_m2_glszm_ZoneEntropy | 0.593 | 0.363 | 0.968 | 0.037 |
|  | lbp_3D_m2_glcm_Imc1 | 2.787 | 1.414 | 5.494 | 0.003 |
|  | gradient_glcm_Correlation | 0.303 | 0.156 | 0.587 | 0.001 |
|  | wavelet_LHL_glcm_DifferenceVariance | 0.578 | 0.344 | 0.973 | 0.039 |
|  | wavelet_LLH_glcm_Correlation | 2.332 | 1.351 | 4.027 | 0.002 |
|  | exponential_glrlm_GrayLevelNonUniformity | 1.864 | 1.034 | 3.36 | 0.038 |
| Combined Model  (UE+AP+VP) | AP_squareroot_firstorder_Skewness | 0.311 | 0.161 | 0.603 | 0.001 |
|  | AP_wavelet_HLH_glcm_Correlation | 0.248 | 0.127 | 0.483 | 0.001 |
|  | AP_wavelet_HLH_firstorder_Mean | 0.261 | 0.124 | 0.553 | 0.001 |
|  | VP_gradient_glcm_Correlation | 2.065 | 1.148 | 3.714 | 0.015 |
|  | VP_lbp_3D_m2_glrlm_LowGrayLevelRunEmphasis | 1.724 | 1.037 | 2.865 | 0.036 |
|  | VP_wavelet_HLH_glcm_Correlation | 0.386 | 0.22 | 0.677 | 0.001 |
|  | UE_wavelet_LLL_glcm_InverseVariance | 2.422 | 1.362 | 4.307 | 0.003 |
|  | UE_wavelet_LLH_gldm_LargeDependenceEmphasis | 0.564 | 0.343 | 0.929 | 0.024 |

**Supplementary Table S2.** Hosmer–Lemeshow Test and DeLong Test Results for Four Radiomics Models, the Pathomics Model, and the Nomogram Model

| Model/Comparison | Hosmer–Lemeshow test | | | Delong test | | | | | |
| --- | --- | --- | --- | --- | --- | --- | --- | --- | --- |
|  | Training | Internal Validation | External  Validation | Training | | Internal Validation | | External  Validation | |
| UE Radiomics | *p* = 0.289 | *p* = 0.757 | *p* = 0.215 |  |  |  |  |  |  |
| AP Radiomics | *p* = 0.158 | *p* = 0.340 | *p* = 0.187 |  |  |  |  |  |  |
| VP Radiomics | *p* = 0.398 | *p* = 0.215 | *p* = 0.389 |  |  |  |  |  |  |
| Combined Radiomics | *p* = 0.187 | *p* = 0.319 | *p* = 0.188 |  |  |  |  |  |  |
| Pathomics | *p* = 0.188 | *p* = 0.805 | *p* = 0.158 |  |  |  |  |  |  |
| Nomogram | *p* = 0.436 | *p* = 0.211 | *p* = 0.331 |  |  |  |  |  |  |
| UE vs AP |  |  |  | *z* =0.912 | *p* = 0.211 | *z* = 1.357 | *p* = 0.318 | *z* = 1.210 | *p* = 0.226 |
| UE vs VP |  |  |  | *z* =1.769 | *p* = 0.047* | *z* = 1.824 | *p* = 0.041* | *z* = 1.950 | *p* = 0.049* |
| AP vs VP |  |  |  | *z* =1.360 | *p* =0.036* | *z* = 1.672 | *p* = 0.047* | *z* = 1.420 | *p* = 0.045* |
| Combined Radiomics vs UE |  |  |  | *z* = -3.035 | *p* = 0.002* | *z* = -3.374 | *p* = 0.007* | *z* = -2.980 | *p* = 0.003* |
| Combined Radiomics vs AP |  |  |  | *z* = -2.949 | *p* = 0.003* | *z* = -2.344 | *p* = 0.019* | *z* = -2.500 | *p* = 0.012* |
| Combined Radiomics vs VP |  |  |  | *z* = -2.818 | *p* = 0.005* | *z* = -2.329 | *p* = 0.020* | *z* = -2.100 | *p* = 0.036* |
| Combined Radiomics vs Pathomics |  |  |  | *z* = - 2.599 | *p* = 0.009* | *z* = 2.323 | *p* =0.020* | *z* = 2.450 | *p* = 0.014* |
| Pathomics vs Nomogram |  |  |  | *z* = -2.500 | *p* = 0.012* | *z* = -3.661 | *p* < 0.001* | *z* = -3.200 | *p* = 0.001* |
| Nomogram vs Combined Radiomics |  |  |  | *z* = -2.217 | *p* = 0.027* | *z* = -3.325 | *p* = 0.001* | *z* =-2.800 | *p* = 0.005* |

Note: p <0.05 and “*” was considered to have a statistical difference.

**Supplementary Table S3.** The selected pathomics features

| Features | OR | 0.025 | 0.975 | *p* value |
| --- | --- | --- | --- | --- |
| Texture_Entropy_Hematoxylin_3_02_256 | 0.491 | 0.224 | 1.074 | 0.075 |
| AreaShape_Zernike_4_4.1 | 2.242 | 1.299 | 3.87 | 0.004 |
| Texture_InfoMeas1_Hematoxylin_3_03_256.1 | 0.273 | 0.112 | 0.664 | 0.004 |

**Supplementary Table S4.** Calibration performance (Brier score and calibration slope) of the Nomogram and single-modality models across the three cohorts

| Model | Training cohort | |  | Internal Validation Cohort | |  | External Validation Cohort | |
| --- | --- | --- | --- | --- | --- | --- | --- | --- |
|  | calibration slope | Brier score |  | calibration slope | Brier score |  | calibration slope | Brier score |
| Combined Radiomics | 0.893 | 0.112 |  | 0.839 | 0.121 |  | 0.816 | 0.128 |
| Pathomics | 0.915 | 0.105 |  | 0.858 | 0.118 |  | 0.832 | 0.125 |
| Nomogram | 0.954 | 0.098 |  | 0.917 | 0.102 |  | 0.887 | 0.110 |

**Supplementary Table S5.** NRI and IDI comparing the Nomogram with single-modality models for predicting MVPs across the three cohorts

| Cohort | Comparison | NRI (95% CI) | *p* value | IDI (95% CI) | *p* value |
| --- | --- | --- | --- | --- | --- |
| **Training cohort** | Nomogram vs. Combined Radiomics | 0.224 (0.035, 0.413) | 0.023 | 0.035 (0.003, 0.067) | 0.032 |
|  | Nomogram vs. Pathomics | **0.298 (0.107, 0.489)** | 0.002 | 0.068 (0.023, 0.113) | 0.009 |
| **Internal Validation Cohort** | Nomogram vs. Combined Radiomics | **0.187 (0.005, 0.369)** | 0.045 | 0.029 (0.001, 0.059) | 0.061 |
|  | Nomogram vs. Pathomics | **0.252 (0.074, 0.430)** | 0.008 | 0.055 (0.014, 0.096) | 0.018 |
| **External Validation Cohort** | Nomogram vs. Combined Radiomics | **0.276 (0.072, 0.480)** | 0.008 | 0.042 (0.009, 0.075) | 0.012 |
|  | Nomogram vs. Pathomics | **0.361 (0.165, 0.557)** | <0.001 | 0.083 (0.037, 0.129) | 0.003 |

**Supplementary Table S6.** The points for each variable in the nomogram and the calculation formula

| Variables | Assignment | Categories | Points |
| --- | --- | --- | --- |
| Rad-score | +20 × (放射组学评分 + 2.5) | | |
| Path-score | +20 × (病理评分 + 2.5) | | |
| Histological Grade | 1 | Grade 1 | 0 |
|  | 2 | Grade 2 | 16.75 |
|  | 3 | Grade 3 | 33.5 |

Points=Rad-score + Path-score + Histological Grade

**Prediction Probability of NAA-MVPs = 1 / (1 + e^(-0.0836 * (Points - 116)))**

*
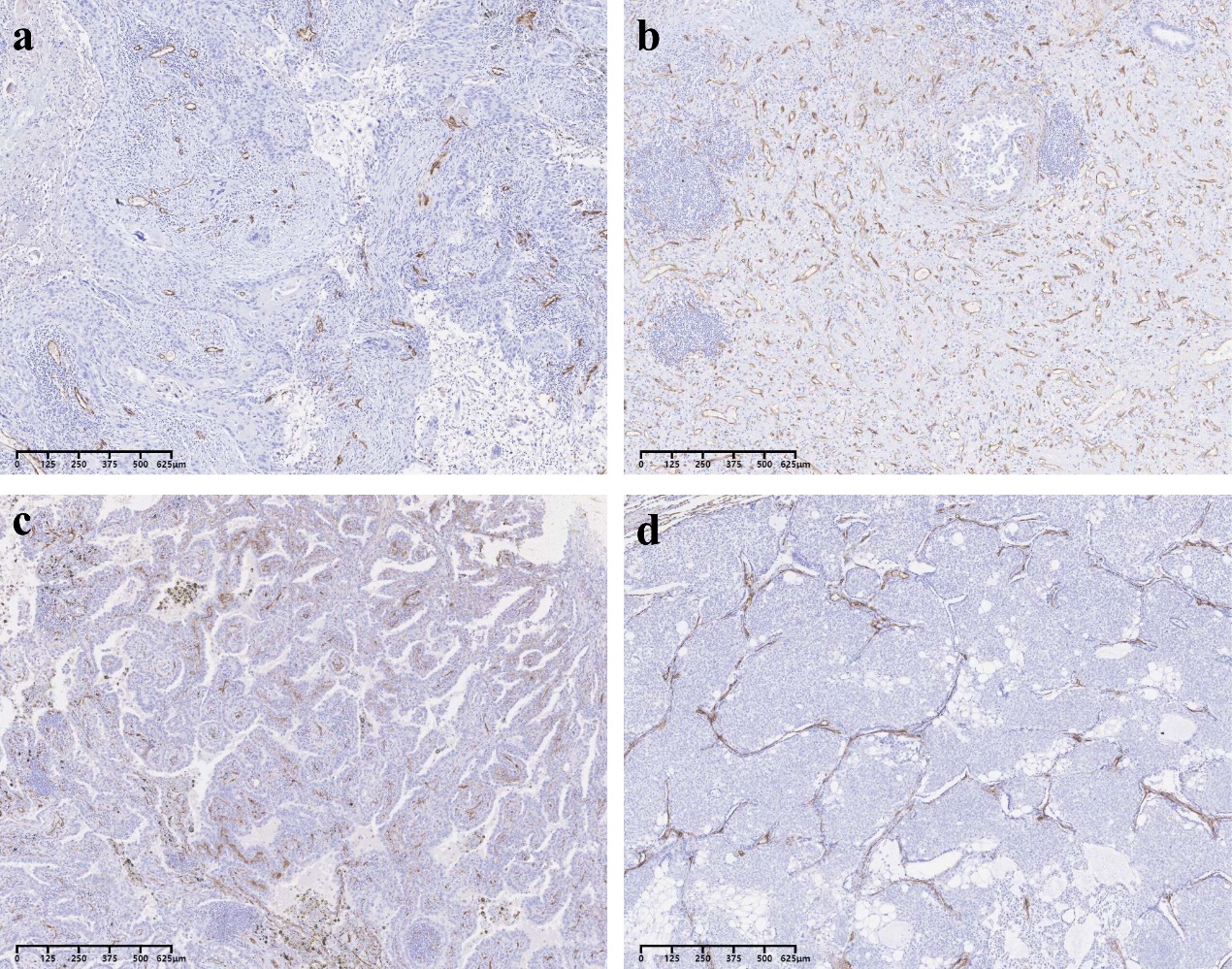
*

**Fig. S1.** Representative CD34-IHC staining patterns demonstrating four angiogenic microvascular patterns (MVPs) in NSCLC tissue. **(a)** Basal angiogenic MVP (BA-MVP): Microvessels are mainly located in the stroma surrounding nests of tumor cells. **(b)** Diffuse angiogenic MVP (DA-MVP): Newly formed vessels, stroma, and tumor cells are diffusely intermingled with loss of normal architecture. **(c)** Papillary angiogenic MVP (PA-MVP): Vessels are present within stromal cores lined by tumor cells, typically forming a single layer. **(d)** Non-angiogenic alveolar MVP (NAA-MVP): Microvessels are confined to alveolar septa with preservation of normal alveolar structures. Brown staining indicates CD34-positive endothelial cells. Scale bar: each division represents 125 μm.


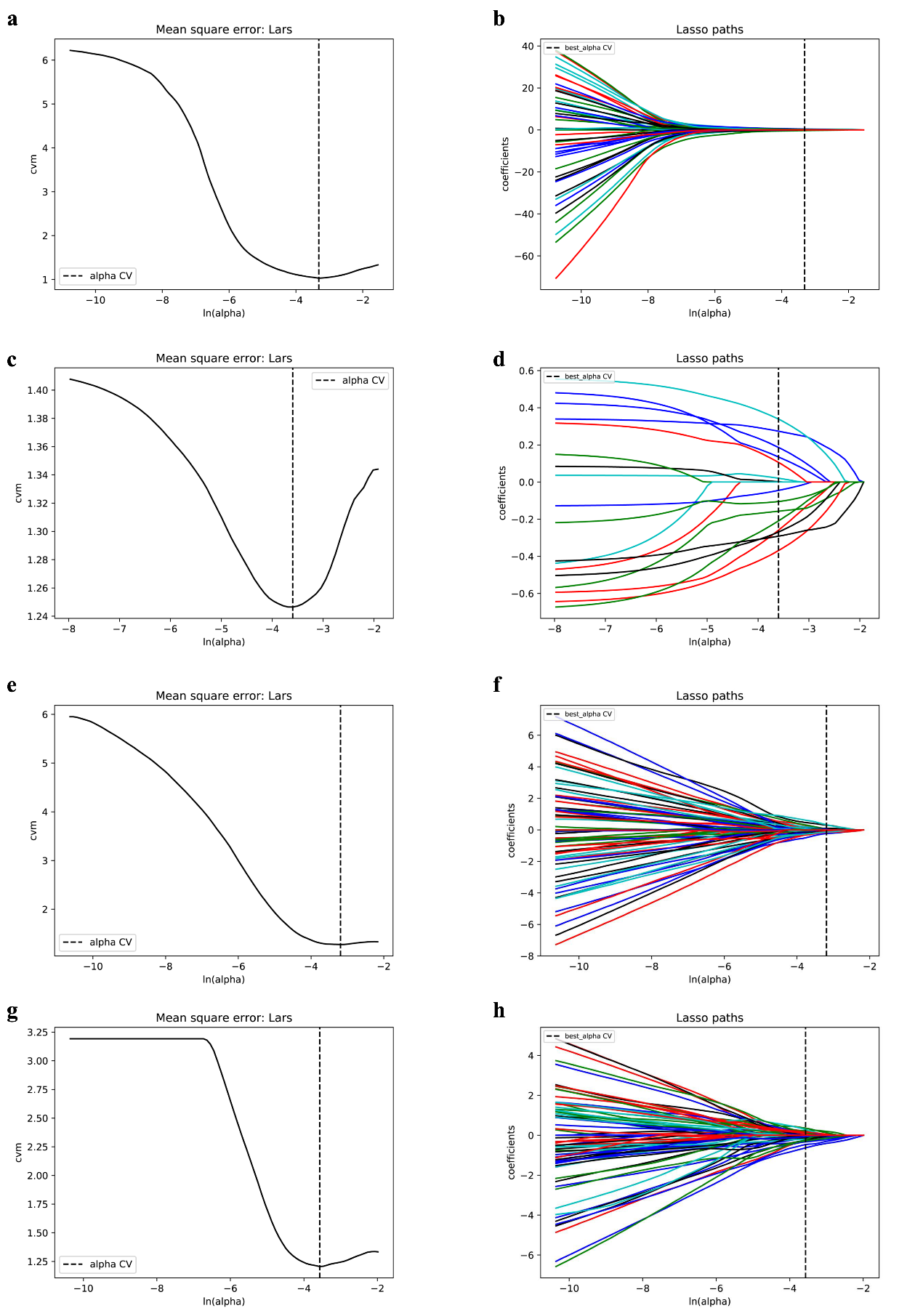


**Fig. S2.** The radiomics features selection process of the Least Absolute Shrinkage and Selection Operator (LASSO) Regression Model, **(a-b)** Feature selection for UE Model, **(c-d)** Feature selection for AP Model, **(e-f)** Feature selection for VP Model, and **(g-h)** for Combined Radiomics Model.


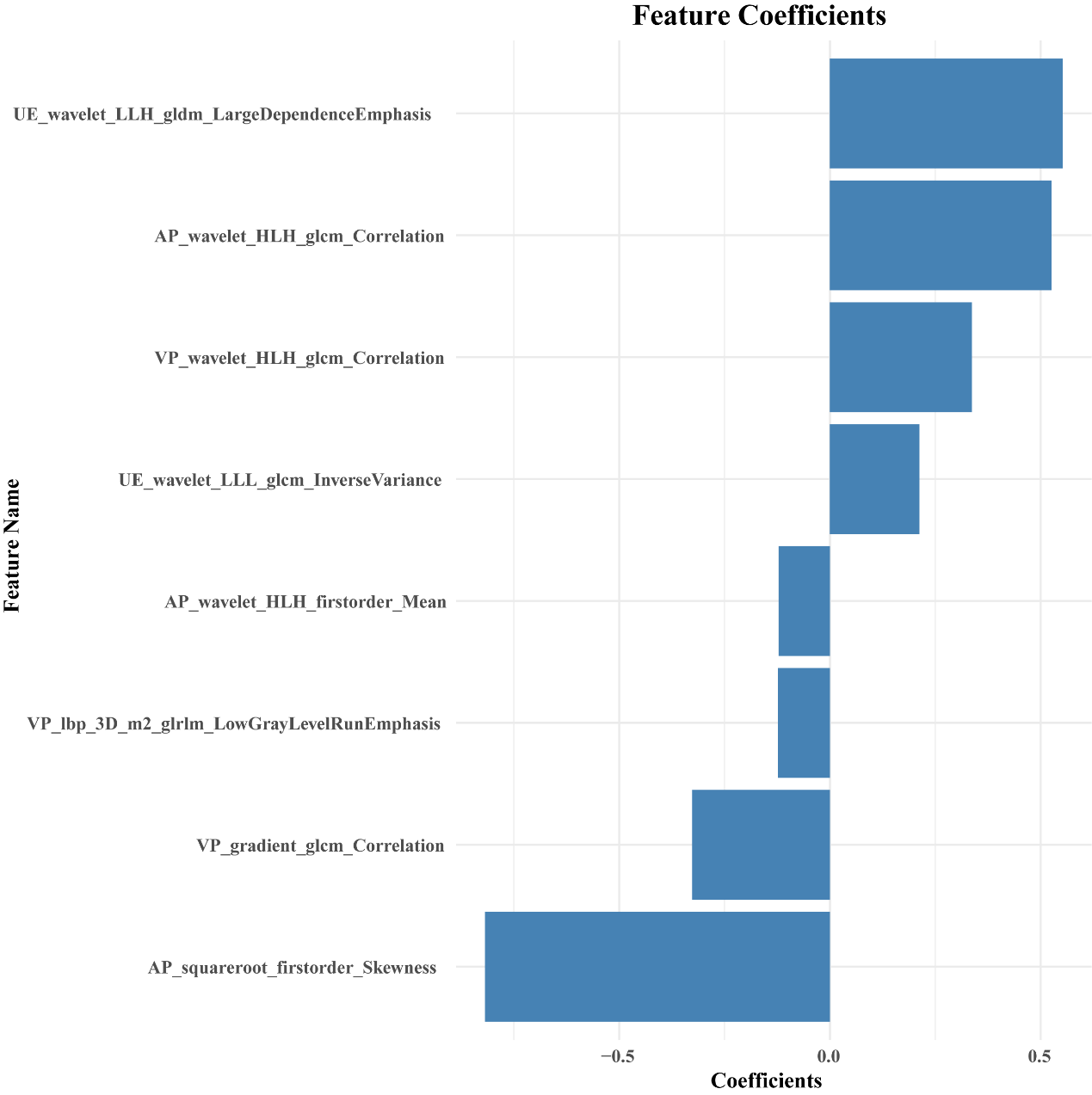


**Fig. S3.** The eight selected radiomic features and corresponding coefficients in the Combined Radiomics Model by the LASSO Regression Algorithm.


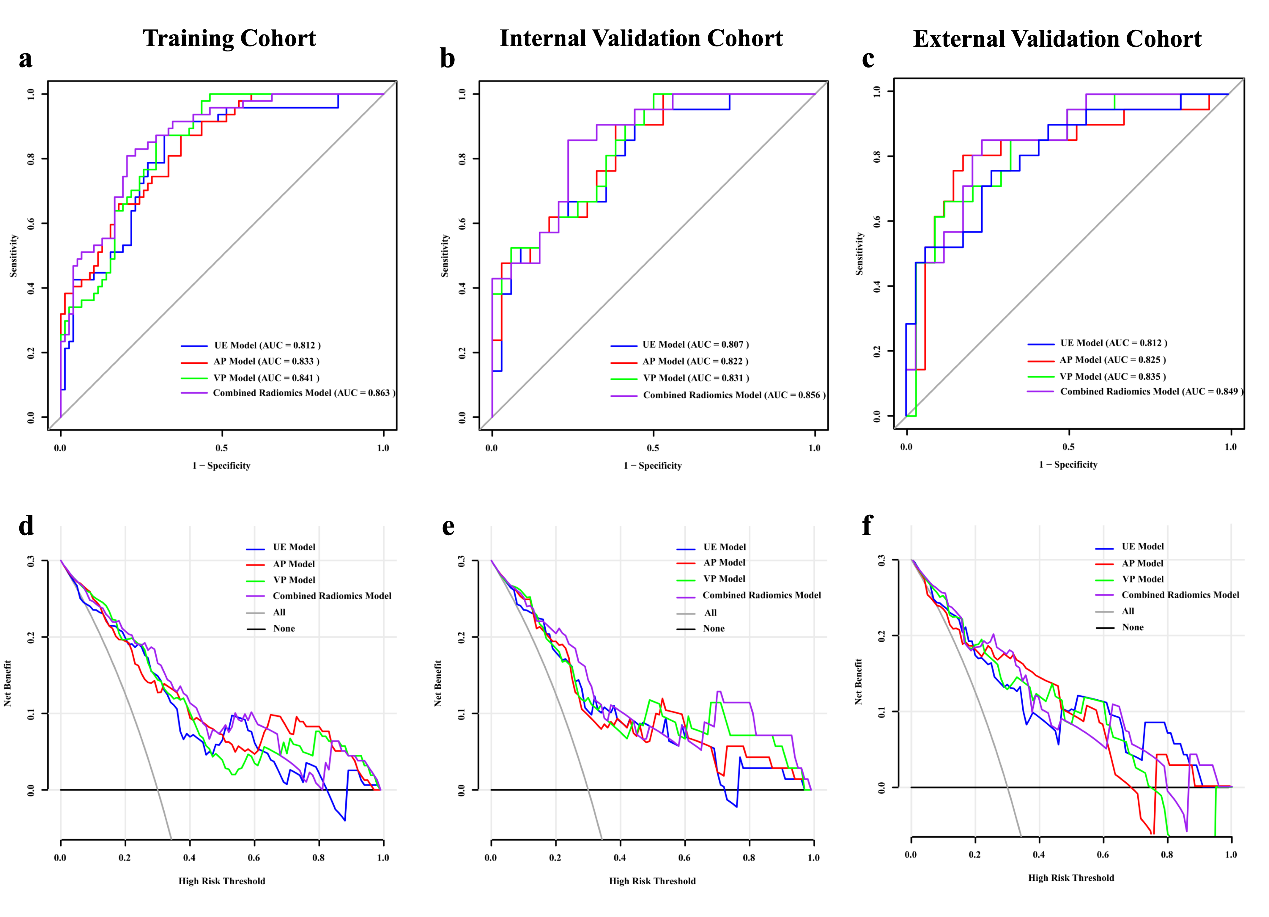


**Fig. S4.** Predictive performance of four radiomics model in the study. **(a)** ROC curves of radiomics models in the training cohort. **(b)** ROC curves of radiomics models in the internal validation cohort. **(c)** ROC curves of radiomics models in the external validation cohort. **(d)** DCA of radiomics models in the training cohort. **(e)** DCA of radiomics models in the internal validation cohort. **(f)** DCA of radiomics models in the external validation cohort. ROC receiver operating characteristic, DCA decision curve analysis.


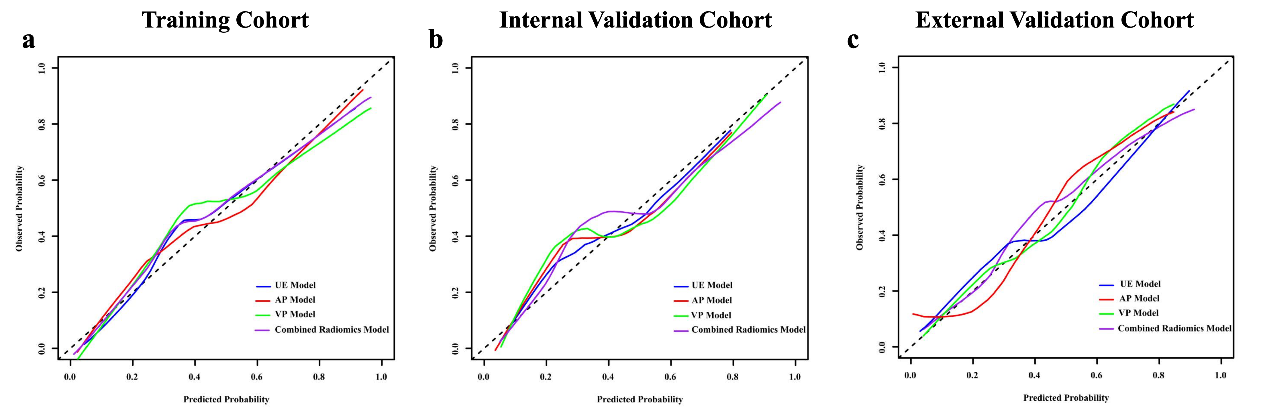


**Fig. S5.** Calibration plots of four radiomics model in the training cohort **(a)**, internal validation cohort **(b)**, and external validation cohort **(c)**.


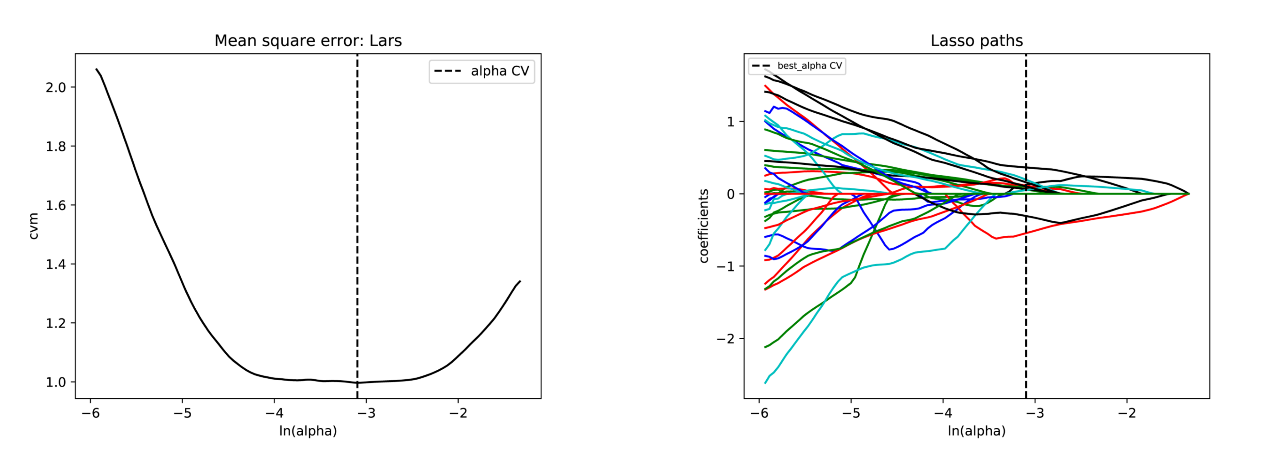


**Fig. S6.** The pathomics features selection process of the Least Absolute Shrinkage and Selection Operator (LASSO) Regression Model.
